# Prevention of Myc‐Driven Prostate Cancer in Mice by Oral Administration of Sulforaphane

**DOI:** 10.1002/mc.70139

**Published:** 2026-06-18

**Authors:** Krishna B. Singh, Eun‐Ryeong Hahm, Bruce L. Jacobs, Shivendra V. Singh

**Affiliations:** ^1^ Department of Pharmacology and Chemical Biology University of Pittsburgh School of Medicine Pittsburgh Pennsylvania USA; ^2^ Division of Health Services Research, Department of Urology University of Pittsburgh School of Medicine Pittsburgh Pennsylvania USA; ^3^ UPMC Hillman Cancer Center University of Pittsburgh Pittsburgh Pennsylvania USA

**Keywords:** fatty acids, prevention, prostate cancer, sulforaphane

## Abstract

Prevention is desirable to reduce suffering and death from prostate cancer. However, a clinical‐grade intervention for the prevention of this malignancy is still lacking. This study was undertaken to determine the feasibility of prostate cancer prevention by broccoli constituent sulforaphane (SFN) because epidemiological studies have suggested an inverse association between intake of broccoli and the risk of prostate cancer. Oral administration of 1 mg SFN/mouse (three times/week) to 5‐week‐old male Hi‐Myc transgenic mice for 5 weeks decreased the incidence of prostatic adenocarcinoma in situ by about 54% without causing weight loss or any other side effects. Existing literature strongly implicates fatty acid synthesis in prostate cancer progression. Therefore, we determined the effect of SFN treatment on fatty acid synthesis. Prostate cancer prevention by SFN in Hi‐Myc mice was accompanied by a decrease in: (a) the uptake of ^11^C‐acetate in the prostate of live mice; (b) plasma levels of fatty acid synthesis intermediates acetyl‐CoA and malonyl‐CoA; (c) circulating levels of total free fatty acids (TFFA); (d) downregulation of fatty acid synthesis enzyme proteins acetyl‐CoA carboxylase 1 and fatty acid synthase; and (e) plasma levels of interkeukin‐10 and interleukin‐12. Because prostate cancer in Hi‐Myc mice is driven by Myc, we determined the effect of overexpression of c‐Myc in 22Rv1 cells on TFFA level. Overexpression of c‐Myc conferred partial protection against TFFA suppression by SFN treatment. In summary, this study reveals that SFN administration prevents prostate cancer development in Hi‐Myc mice by suppressing fatty acid synthesis.

**Abbreviations**
ACC1: acetyl‐CoA carboxylase 1
ANOVA: analysis of variance
FASN: fatty acid synthase
HG‐PIN: high‐grade prostatic intraepithelial neoplasia
LG‐PIN: low‐grade prostatic intraepithelial neoplasia
PAC: prostate adenocarcinoma in situ
PBS: phosphate‐buffered saline
PET: positron emission tomography
ROI: region of interest
SFN: sulforaphane
SMA: smooth muscle actin
SUV: standardized uptake value
TFFA: total free fatty acids
TRAMP: transgenic adenocarcinoma of the mouse prostate
TUNEL: terminal deoxynucleotidyl transferase dUTP nick end labeling

## 1 Introduction

Prostate cancer continues to be the second leading cause of cancer‐related deaths in American men [1]. Diagnosis of about 333,830 new prostate cancer cases is expected in 2026, according to the American Cancer Society. From year 2014 through 2022, the incidence rate of prostate cancer increased by 3% per year. In the year 2026, about 36,320 American men are expected to die from this disease. Epidemiological studies have identified several risk factors for prostate cancer, including age, ethnicity, family history, insulin‐like growth factors, lifestyle, diet, and environmental and occupational exposures [2]. Because many risk factors for prostate cancer (e.g., age, race, family history) are not modifiable, prevention using synthetic small molecules, natural phytochemicals, and biological molecules like vaccines is attractive for reducing death from this malignancy [3]. Three large randomized clinical trials have tested the feasibility of prostate cancer prevention using 5α‐reductase inhibitors and selenium + vitamin E combination [4, 5, 6]. The 5α‐reductase inhibitors finasteride and dutasteride were not approved for prostate cancer prevention because of high‐grade tumors in the inhibitor arms when compared to the placebo group [4, 5]. Primary prevention of prostate cancer was not achieved with the selenium + vitamin E combination in the SELECT trial [6].

Medicinal and dietary plants or their bioactive components are attractive for the prevention of prostate cancer [7, 8]. Broccoli constituent sulforaphane (SFN) is one such bioactive phytochemical that appears promising for the prevention of prostate cancer [9]. SFN is stored in broccoli as a glucosinolate (glucoraphanin). Glucoraphanin is converted to SFN upon cutting or chewing of broccoli [10]. Gut microbiome can also facilitate glucoraphanin breakdown to SFN [11]. Preclinical studies have revealed that SFN inhibits the growth of human prostate cancer cells in vitro by causing G_2_/M phase cell cycle arrest and apoptotic cell death [12, 13]. On the other hand, a normal prostate epithelial cell line (PrEC) was resistant to apoptosis induction by SFN [13]. Cancer cell selectivity is highly desirable for cancer preventive agents. Apoptosis induction by SFN was mediated by Bax and Bak, leading to caspase‐3 activation [13, 14]. Self‐renewal of prostate cancer stem‐like cells was also inhibited by SFN treatment [15]. SFN induces cytoprotective autophagy in PC‐3 and LNCaP human prostate cancer cells [16]. Treatment of PC‐3 cells with the autophagy inhibitor 3‐methyladenine exacerbated SFN‐mediated release of cytochrome *c* as well as apoptotic cell death [16].

We were the first to report that oral administration of SFN (5.6 µmol, 3 times/week) significantly inhibited growth of PC‐3 xenografts in nude mice without causing any side effects [14]. For example, the average tumor volumes in control and SFN‐treated mice were 170 ± 13 and 80 ± 14 mm^3^, respectively, reflecting a 53% reduction in tumor volume after SFN treatment [14]. Our observation of PC‐3 xenograft growth inhibition by SFN was independently confirmed by other investigators [17]. SFN was bioavailable in the mouse prostate after oral administration of 5 and 20 µmoles [18]. SFN metabolites were also detectable in human prostate biopsies after daily oral intake of broccoli sprout extract [19]. Oral gavage of 6 µmol SFN (three times/week) reduced the incidence of the prostatic intraepithelial neoplasia (PIN) and well‐differentiated carcinoma in Transgenic Adenocarcinoma of the Mouse Prostate (TRAMP) mice by about 23% and 28%, respectively, in the dorsolateral prostate [20]. The SFN‐treated mice exhibited about 50% and 63% decrease in pulmonary metastasis incidence and multiplicity compared with control mice [20]. In this study, we used a human‐relevant transgenic mouse model (Hi‐Myc) to determine prostate cancer prevention after oral administration of SFN. Prostate cancer in Hi‐Myc mice shares molecular resemblance with human prostate cancer [21]. Gene expression profiling from mouse and human prostate tumors identified a *Myc* expression signature that included *NKX3.1* and Pim‐1 kinase [22]. Pim‐1 kinase is known to cooperate with Myc to promote prostate cancer [22].

## 2 Materials and Methods

### 2.1 Reagents

SFN (purity ≥ 98%) was purchased from LKT laboratories (St. Paul, MN). A stock solution of SFN was prepared in dimethyl sulfoxide and diluted with culture medium prior to use. Cell culture reagents were purchased from Life Technologies Thermo‐Fisher Scientific (Waltham, MA) or Corning (Manassas, VA). Anti‐acetyl‐CoA carboxylase 1 (ACC1) antibody (cat. # 21923; RRID: AB_11042445) was purchased from ProteinTech (Rosemont, IL), and anti‐fatty acid synthase (FASN) antibody (cat. # 3180; RRID:AB_2100796) was purchased from Cell Signaling Technology (Beverly, MA). Anti‐c‐Myc (cat. # ab39688; RRID:AB_731661) antibody was purchased from Abcam (Cambridge, MA). Anti‐α‐smooth muscle actin (α‐SMA) (cat. # sc‐32251; RRID:AB_262054) antibody was purchased from Santa Cruz Biotechnology (Dallas, TX). Anti‐Ki‐67 antibody was from Abcam (cat. # ab15580). Kits for the determination of acetyl‐CoA (cat. # K317‐100), cholesterol, and cholesteryl ester (cat. # K603‐100) were purchased from BioVision (Milpitas, CA). Kits for the determination of total free fatty acids (TFFA) (cat. # MAK044) and total phospholipids (cat. # MAK122) were purchased from Sigma‐Aldrich (St. Louis, MO). ELISA kit for mouse malonyl‐Co A was purchased from MyBiosource (San Diego, USA).

### 2.2 Chemoprevention Study

Male transgenic Hi‐Myc mice [FVB‐Tg (ARR2/Pbsn‐MYC)] were obtained from the National Cancer Institute Mouse Repository and were crossed with wild‐type female FVB/N mice to obtain male Hi‐Myc mice. Mice were fed a standard diet provided by the animal facility and were housed under a 12 h light/12 h dark cycle. After transgene verification, 5‐week‐old male Hi‐Myc transgenic mice were randomly divided into two groups. The control group of mice was administered by oral gavage of 100 µL corn oil, while the second group of mice was administered with oral gavage of 1 mg SFN/mouse in 100 µL corn oil three times/week for 5 weeks. At the end of the study, blood and prostate tissues were collected. The urogenital system was harvested, and the urinary bladder and seminal vesicles were removed. The entire prostate gland was collected without separating the individual lobes and fixed in 10% neutral‐buffered formalin. Pathological evaluation was performed for all lobes. Plasma samples were aliquoted and stored at −80°C for biochemical analysis. Body weights of mice were recorded weekly.

### 2.3 ^11^C‐Acetate Positron Emission Tomography (PET)

PET imaging was done using Siemens Eclipse HP11 MeV imager. Animals were given 600 µCi of ^11^C‐acetate via lateral tail vein in a single bolus. Imaging was done at 20 min post‐injection to allow the tracer to circulate. PET acquisition protocol was an 8‐min static scan. Standard low‐resolution computed tomography for anatomic reference was performed after PET acquisition. The region of interest (ROI) analysis was performed by drawing multiple slice ROI for the prostate and interpolating to form a volumetric ROI. Prostate standard uptake volume (SUV) was normalized to muscle to obtain an SUV ratio. The SUV ratio was compared between groups and analyzed using *t*‐test.

### 2.4 Measurement of Lipid Metabolites

Levels of acetyl‐CoA, malonyl‐CoA, TFFA, total phospholipids, cholesterol, and cholesteryl ester in the plasma of control and SFN‐treated Hi‐Myc mice were determined using commercially available kits, and by following the manufacturer's protocol. Due to mixed pathology, including low‐grade (LG)‐PIN, high‐grade PIN (HG‐PIN), and prostate adenocarcinoma in situ (PAC), metabolite levels were not measured in the prostate tissue.

### 2.5 Immunohistochemistry

Prostate sections (4–5 μm thick) were de‐paraffinized, hydrated, washed with phosphate‐buffered saline (PBS), and incubated in citrate retrieval buffer solution (pH 6.0) for 20 min at 100°C followed by washing with PBS. Sections were treated with blocking buffer for 1 h followed by incubation with primary antibodies (anti‐ACC1: 1:500 dilution; anti‐FASN: 1:500 dilution; and anti‐α‐SMA: 1:1000 dilution) overnight in humidified chambers at 4°C. Sections were washed with PBS and incubated with Alexa Fluor 488 and/or Alexa Fluor 568‐conjugated or Alexa Fluor 633‐conjugated secondary antibody for 1–2 h at room temperature or horseradish peroxidase‐conjugated secondary antibody for 2 h at room temperature. For the fluorescence‐based immunohistochemistry, sections were washed with PBS after secondary antibody incubation and mounted in media containing 4′,6‐diamidino‐2‐phenylindole. Stained sections were examined under Nikon A1 confocal microscope, and protein expression intensity was analyzed using ImageJ software.

### 2.6 Terminal Deoxynucleotidyl Transferase‐Mediated dUTP Nick End Labeling (TUNEL) Assay for Apoptosis Detection

TUNEL assay was performed using a kit from Millipore Sigma (cat. # S7110). Paraffin‐embedded prostate tissue sections from control and SFN‐treated mice were deparaffinized by xylene and serially washed with absolute ethanol, 95% ethanol, 70% ethanol, and PBS. Freshly prepared proteinase K (20 µg/mL) was applied directly to the sections for 15 min at room temperature. After repeated washing with PBS, equilibrium buffer (supplied with the kit) was applied for 10 s and then the TdT enzyme (supplied with the kit) was added to the sections. The sections were then incubated in a humidified chamber for 60 min at 37°C. Next, the slides were placed in a jar containing stop/wash buffer (supplied with the kit) for 15 s and washed with PBS. The next step was to treat the sections at room temperature with digoxigenin conjugate followed by incubation at room temperature for 30 min. After washing with PBS, the sections were mounted on a glass coverslip in 4′,6‐diamidino‐2‐phenylindole containing mounting medium. Stained sections were examined under a Leica DM microscope equipped with DFC 450 C digital camera at ×200 magnification. The number of TUNEL‐positive cells per high‐power field was counted manually from 10 randomly selected non‐overlapping fields.

### 2.7 Measurement of Plasma Cytokines

Fluorokine MAP cytokine multiplex kit from Millipore was used (catalog # NC1074016/MCYTOMAG‐70K). Cytokines levels in the plasma from Hi‐Myc mice were determined using Luminex technology. Luminex Multiplex Bead Immunoassays are solid phase sandwich immunoassays. Spectral properties of 100 distinct bead regions can be monitored with the Luminex 100/200 instrument. Beads of defined spectral properties conjugated to analyte‐specific capture antibodies are placed into the wells of a filter bottom microplate and incubated at room temperature for 120 min. During this first incubation, analytes bind to the capture antibodies on the beads. After washing with PBS, analyte‐specific biotinylated detector antibodies were then added and incubated at room temperature for 60 min. During this second incubation, the analyte‐specific biotinylated detector antibodies recognize their epitopes and bind to the appropriate immobilized analytes. Streptavidin conjugated to the fluorescent protein (R‐Phycoerythrin) was added and the samples were incubated at room temperature for 30 min. After washing to remove unbound Streptavidin‐R‐Phycoerythrin and biotinylated detector antibodies, the beads were analyzed with the Luminex 100/200 instrument.

### 2.8 Cell Lines

22Rv1 cell line (RRID: CVCL_1045) was purchased from the American Type Culture Collection (Manassas, VA) and cultured as recommended by the supplier. The cell line was authenticated by us in March of 2017 by IDEXX Laboratories. Cells from authenticated stocks were used for experiments. The cells were stably transfected with empty pcDNA3 vector or pcDNA3‐Myc plasmid.

### 2.9 Immunoblotting

Cells were treated with solvent (control) or 5 or 10 µM of SFN for 24 h and lysed as described by us previously [23]. Immunoblotting was performed as described by us previously [23]. Immunoreactive bands were visualized by the enhanced chemiluminescence method. Densitometric quantitation was performed with the use of UN‐SCAN‐IT version 5.1 (Silk Scientific Corporation, Orem, UT).

### 2.10 Statistical Analyses

Statistical significance of difference was determined by Student's *t*‐test or one‐way analysis of variance (ANOVA) followed by Bonferroni's (multiple comparison) test using GraphPad Prism (version 8.0.0).

## 3 Results

### 3.1 Oral Administration of SFN Prevents Prostate Cancer Development in Hi‐Myc Mice

**Figure 1:**
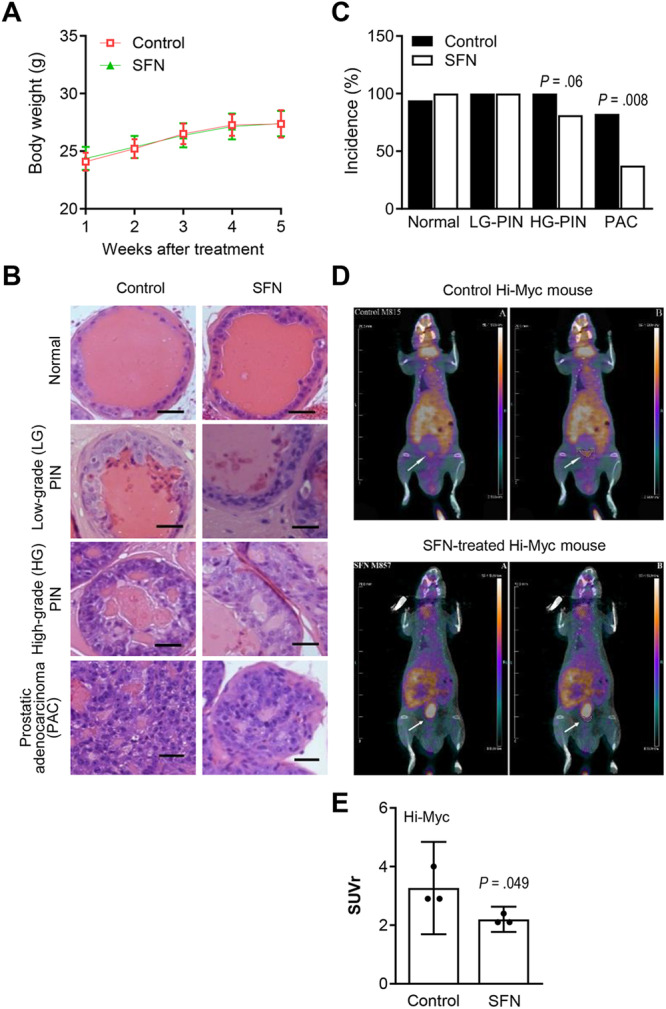
Oral administration of sulforaphane (SFN) prevents prostate cancer development in Hi‐Myc mice. (A) Body weight of mice over time in the vehicle and SFN treatment groups. Results shown are mean ± 95% CI (*n* = 18 for control; *n* = 17 for SFN group). Statistical difference was determined by Student's *t*‐test. (B) Representative hematoxylin and eosin‐stained prostate tissue sections from control and SFN‐treated mice (20× objective magnification, scale bar = 50 µm). (C) Incidence of low‐grade prostatic intraepithelial neoplasia (LG‐PIN), high‐grade PIN (HG‐PIN), and prostatic adenocarcinoma in situ (PAC) in control and SFN‐treated mice. The results shown are mean ± 95% CI (*n* = 17 for control; *n* = 16 for SFN group). Statistical significance was determined by Student's *t*‐test. (D) ^11^C‐acetate PET images of a control mouse and an SFN‐treated mouse. Arrow denotes ventral prostate area. (E) Quantitation of ^11^C‐acetate uptake in the prostate. Prostate SUVs were normalized to muscle to obtain an SUV ratio (SUV_r_). Results shown are mean ± 95% CI (*n* = 3). Statistical significance was determined by Student's *t*‐test.

SFN administration did not cause weight loss (Figure 1A). Figure 1B shows histology of the normal prostate gland, LG‐PIN, HG‐PIN, and PAC. The incidence of HG‐PIN was lower by about 19% in the SFN treatment group when compared to the control group, but the difference was not statistically significant (Figure 1C). On the other hand, oral administration of SFN decreased the incidence of PAC by about 54% when compared to the control group (Figure 1C). These results indicate that SFN administration inhibits progression from PIN to PAC in Hi‐Myc mice.

### 3.2 Oral Administration of SFN Inhibits Prostate Uptake of ^11^C‐Acetate

We have shown previously that prostate cancer prevention by SFN administration in TRAMP mice is associated with suppression of fatty acid synthesis, which is elevated in human prostate cancer and represents a valid target for prevention [24, 25]. We raised the question of whether this association was unique to the TRAMP model. We determined the effect of SFN administration on fatty acid synthesis in live mice using ^11^C‐acetate PET. ^11^C‐Acetate is incorporated into acetyl‐CoA, which is an intermediate in fatty acid synthesis [25, 26]. PET images for control and SFN‐treated mice are shown in Figure 1D. Uptake of ^11^_C_‐acetate in the prostate of SFN‐treated mice was lower by about 33% when compared to control mice (Figure 1E). These results suggested that SFN treatment might inhibit fatty acid synthesis in Hi‐Myc mice.

### 3.3 Effect of SFN Administration on Plasma Levels of Fatty Acid Synthesis Intermediates

**Figure 2:**
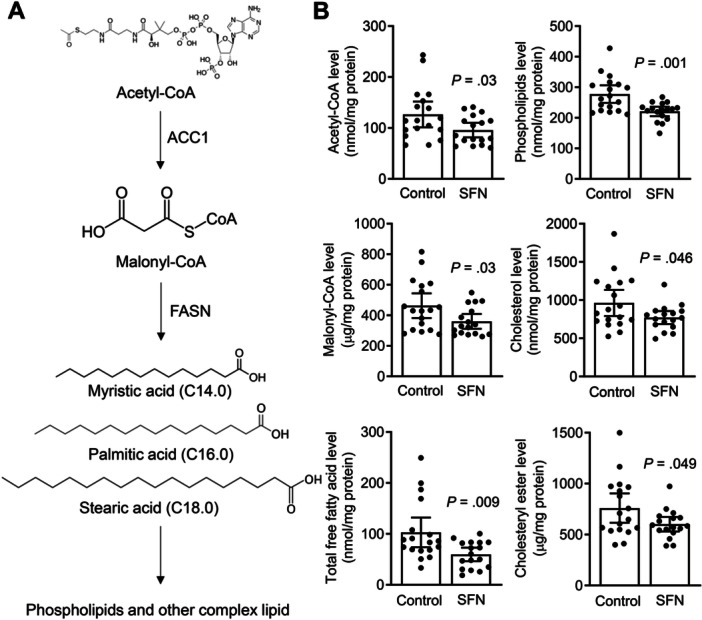
Effect of sulforaphane (SFN) administration on plasma metabolite levels. (A) Fatty acid synthesis pathway. (B) Plasma levels of acetyl‐CoA, malonyl‐CoA, total free fatty acids, total phospholipids, total cholesterol, and cholesteryl ester. Results shown are mean ± 95% CI (*n* = 18 for control and *n* = 17 for SFN group). Statistical analysis was performed by Student's *t*‐test.

Figure 2A summarizes biochemical steps in the fatty acid synthesis pathway. Plasma level of acetyl‐CoA was lower by about 25% in the SFN treatment group when compared to the control, and this difference was statistically significant with a *p* value of 0.03 (Figure 2B). Oral administration of SFN decreased plasma level of malonyl‐CoA by about 22% (*p *= 0.03 compared with the control group) (Figure 2B). Plasma level of TFFA, which includes saturated fatty acids C16:0 palmitic acid, C14:0 myristic acid, and C18:0 stearic acid, was decreased by about 42% after SFN administration (*p *= 0.009 compared to control group) (Figure 2B). Saturated fatty acids are converted to phospholipids. Oral administration of SFN resulted in about 20% decrease in plasma levels of phospholipids (*p *= 0.001 compared to control) (Figure 2B). Acetyl‐CoA is also utilized for the biosynthesis of cholesterol. Plasma level of cholesterol was decreased by about 20% in the SFN treatment group when compared to control (*p *= 0.046 compared to control) (Figure 2B). Oral administration of SFN resulted in about 21% decrease in plasma levels of cholesteryl ester (*p *= 0.049 compared to control group) (Figure 2B). Because of mixed histology in the prostate (normal, LG‐PIN, HG‐PIN, and PAC), prostate levels of metabolites were not measured.

### 3.4 Oral Administration of SFN Downregulates Expression of Fatty Acid Synthesis Enzyme Protein in the Prostate of Hi‐Myc Mice

**Figure 3:**
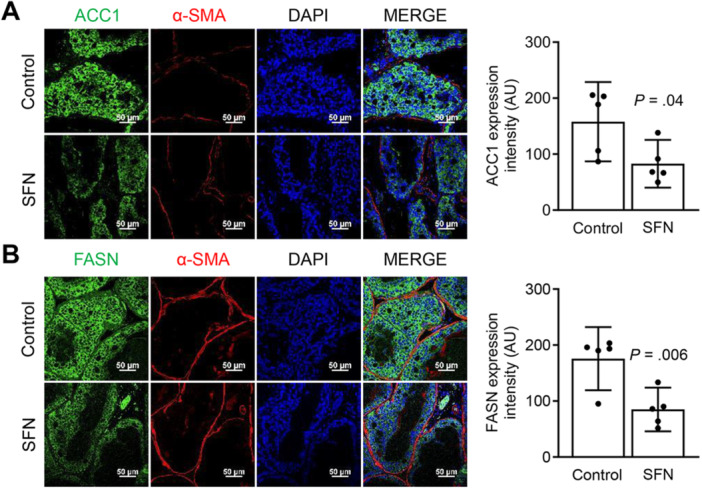
Effect of sulforaphane (SFN) treatment on expression of fatty acid synthesis enzyme proteins. Representative immunohistochemical images for (A) acetyl‐CoA carboxylase 1 (ACC1) and (B) fatty acid synthase (FASN) in prostate tissues of control and SFN‐treated Hi‐Myc mice (40× oil objective magnification; scale—50 μm). Sections were stained with a stromal marker α‐smooth muscle actin (SMA) protein (red fluorescence). Nuclei were visualized by staining with 4′,6‐diamidino‐2‐phenylindole (DAPI). Right panels show quantitation of ACC1 and FASN protein expression using ImageJ software. Results shown are mean ± 95% CI (*n* = 5). Statistical significance was determined by Student's *t*‐test. AU, arbitrary unit.

Figure 3A depicts immunohistochemical images for the expression of ACC1 protein, which catalyzes the conversion of acetyl‐CoA to malonyl‐CoA. Expression of ACC1 protein in the prostate was decreased by about 48% upon SFN administration (*p* = 0.04 compared to the control group). Acetyl‐CoA and malonyl‐CoA are utilized to produce saturated fatty acids through catalytic mediation of FASN. Oral SFN administration decreased expression of FASN protein by about 52% when compared to the control group (*p *= 0.006) (Figure 3B). These results explain SFN‐mediated decrease in circulating levels of TFFA.

### 3.5 SFN Administration Decreases Cell Proliferation and Induces Apoptosis in the Prostate of Hi‐Myc Mice

**Figure 4:**
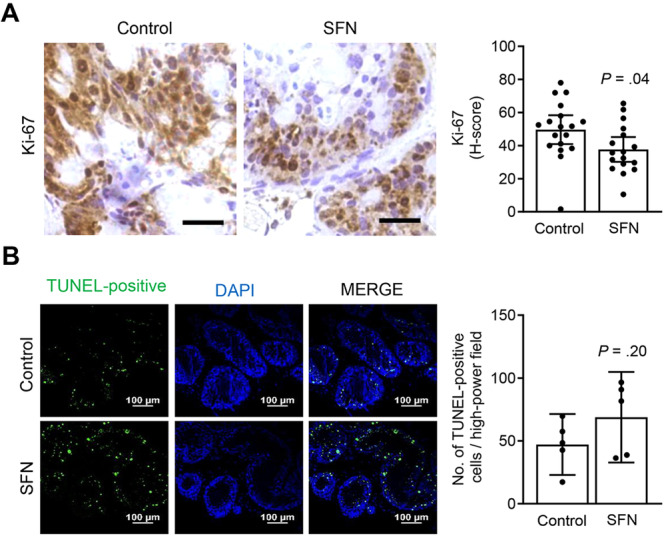
Effect of sulforaphane (SFN) administration on cell proliferation and apoptosis. (A) Representative immunohistochemical images for Ki‐67 (20× objective magnification, scale—50 µm) in prostate tissues from control and SFN‐treated mice. Right panel shows quantification of Ki‐67 expression. The results shown are mean ± 95% CI (*n* = 18 for control; *n* = 17 for SFN group). Statistical significance was determined by Student's *t*‐test. (B) Representative images showing TUNEL‐positive apoptotic cells (20× objective magnification, scale bar = 100 µm) in prostate tissues from control and SFN‐treated mice. Right panel shows quantitation of TUNEL‐positive apoptotic cells per high‐power field. Results shown are mean ± 95% CI (*n* = 5). Statistical significance was determined by Student's *t*‐test.

Genetic or pharmacological suppression of ACC1 or FASN inhibits cell proliferation and/or induces apoptosis [27, 28, 29, 30]. Therefore, we determined the effect of SFN administration on cell proliferation and apoptosis by immunohistochemistry for Ki‐67 and TUNEL assay, respectively. The H‐score for Ki‐67 expression in the prostate of SFN‐treated mice was lower by about 24% when compared to control (*p *= 0.04) (Figure 4A). There was about 1.5‐fold increase in the number of TUNEL‐positive cells in the prostate of SFN‐treated mice compared to control mice, but the difference did not reach statistical significance due to large data scatter (Figure 4B). These results indicate that prostate cancer prevention by SFN treatment was accompanied by inhibition of cell proliferation.

### 3.6 SFN Administration Decreases Plasma Levels of Interleukin IL‐10 and IL‐12

**Figure 5:**
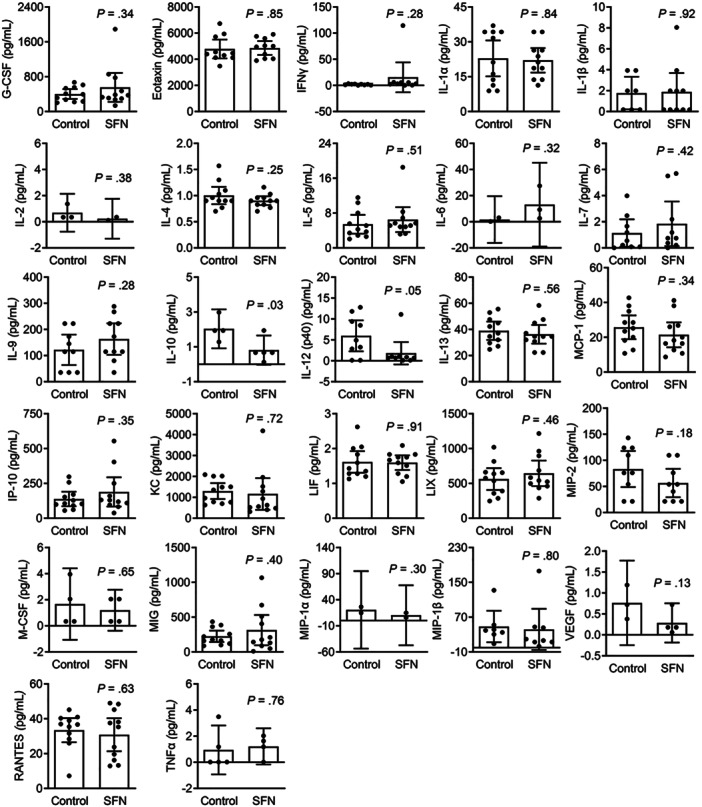
Effect of sulforaphane (SFN) administration on plasma levels of cytokines. Results shown are mean ± 95% CI (*n* = 2–11). Statistical significance was determined by Student's *t*‐test.

Cytokines play an important role in prostate cancer progression by affecting growth, angiogenesis, endothelial mesenchymal transition, etc. [31]. Plasma levels of IL‐10 and IL‐12 were decreased significantly after SFN administration (Figure 5).

### 3.7 Myc Overexpression Partially Protected Against SFN‐Mediated Suppression of TFFA

**Figure 6:**
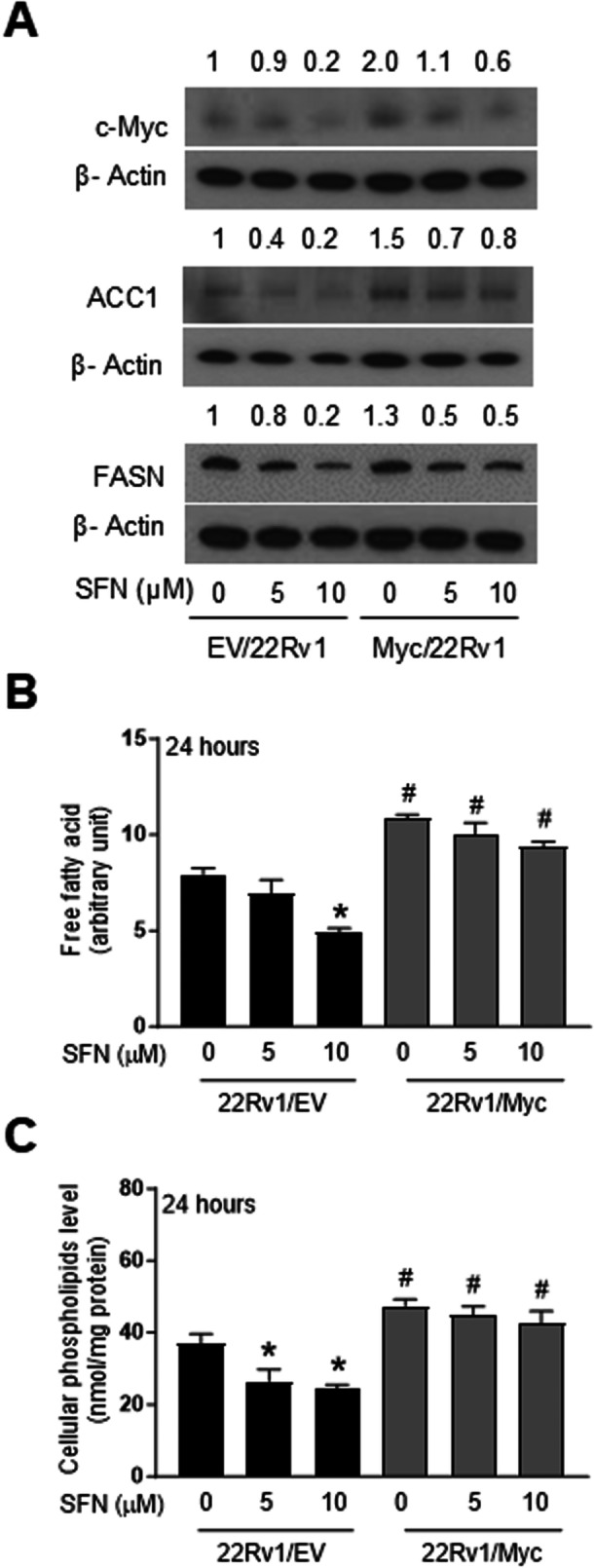
Effect of Myc overexpression on sulforaphane (SFN)‐mediated decrease in protein levels of acetyl‐CoA carboxylase 1 (ACC1) and fatty acid synthase (FASN). (A) Immunoblotting for c‐Myc, ACC1, and FASN using lysates from 22Rv1 cells stably transfected with empty vector (EV) or Myc plasmids and treated for 24 h with dimethyl sulfoxide (DMSO) or the indicated doses of SFN. Intracellular levels of (B) total free fatty acids and (C) total phospholipids in 22Rv1 cells stably transfected with EV or Myc plasmids and treated for 24 h with DMSO or the indicated doses of SFN. Results shown are mean ± SD (*n* = 2–3). Statistically significant (*p* < 0.05) compared with the *corresponding DMSO‐treated control or ^#^between cells transfected with EV and Myc by one‐way ANOVA followed by Bonferroni's multiple comparisons test. Each experiment was repeated at least two times.

We have shown previously that Myc is a direct regulator of ACC1 and FASN expression in human prostate cancer cells [32]. Stable transfection of 22Rv1 cells with Myc plasmid (22Rv1/Myc cells) resulted in twofold overexpression of the protein when compared to empty vector‐transfected control cells (22Rv1/EV cells). SFN‐mediated downregulation of ACC1 and FASN proteins was partially attenuated by Myc overexpression in 22Rv1 cells (Figure 6A). For example, expression of FASN protein was decreased by about 80% upon treatment of 22Rv1/EV cells with 10 µM SFN (Figure 6A). A decrease of only 50% in FASN protein expression was observed by a similar SFN treatment in 22Rv1/Myc cells (Figure 6A). Myc overexpression also conferred protection against SFN‐mediated suppression of TFFA and phospholipid levels (Figure 6B,C). For example, the TFFA level was decreased by about 38% upon treatment of 22Rv1/EV cells with 10 µM SFN (Figure 6B). A decrease of only 13% in TFFA level was observed by a similar SFN treatment in 22Rv1/Myc cells (Figure 6B). The level of phospholipids was decreased by about 34% upon treatment of 22Rv1/EV cells with 10 µM SFN (Figure 6C). A decrease of only about 10% in phospholipid level was observed by a similar SFN treatment in 22Rv1/Myc cells (Figure 6C). These results indicate that suppression of TFFA and phospholipids by SFN treatment is partly mediated by downregulation of Myc.

## 4 Discussion

The present study reveals that prostate cancer prevention by SFN in Hi‐Myc mice is associated with suppression of TFFA levels. SFN‐mediated downregulation of ACC1, which is the rate‐limiting enzyme in the fatty acid synthesis pathway, and FASN, which is responsible for the synthesis of saturated fatty acids, proteins, explains the suppression of TFFA levels. Despite downregulation of ACC1 and FASN proteins in the prostate of Hi‐Myc mice by oral SFN treatment, circulating levels of fatty acid synthesis intermediates and TFFA are only modestly decreased. However, a modest decrease in metabolite level can be clinically important. For example, a blood cholesterol level below 200 mg/dL is considered normal. A 33% higher level of cholesterol is considered very high and a risk factor for cardiovascular disease. Similarly, a normal fasting blood glucose level is 70–99 mg/dL, whereas a fasting blood glucose of 150 is an indicator of diabetes.

The fatty acid synthesis pathway is a valid target for the prevention and treatment of prostate cancer. In the NIH‐AARP Diet and Health Study involving 288,268 men and 23,281 prostate cancer patients and 9 years of follow‐up, higher intake of saturated fatty acids increased the risk of prostate cancer [33]. In the SABOR (San Antonio Biomarkers of Risk) cohort study covering a total of 1903 men of whom 229 had prostate cancer, a median follow‐up of 8.9 years revealed that the relative risk of prostate cancer was associated with intake of dietary fat, especially saturated fatty acids [34]. Overexpression of ACC1 and FASN has been reported in human prostate cancer [32, 35]. Despite concerted efforts from scientists in academia and pharmaceutical industry, a clinically useful inhibitor of fatty acid synthesis is still lacking. Several inhibitors of FASN (e.g., C75, cerulenin, orlistat, etc.) have been identified, but none of them is approved for cancer treatment [36]. Small molecules derived from dietary or medicinal plants were shown to decrease expression of FASN and/or lower TFFA level in prostate cancer cells in vitro, including green tea component epigallocatechin‐3‐gallate and cruciferous vegetable component phenethyl isothiocyanate [37, 38]. However, a randomized controlled clinical trial with green tea failed to show downregulation of FASN expression in prostate cancer cells [39].

The present study reveals that prostate cancer prevention by SFN is associated with a decrease in circulating levels of IL‐10 and IL‐12. The role of IL‐10 in prostate cancer is controversial. Expressions of IL‐6, IL‐10, and heat shock protein 90 were analyzed in 168 prostatic carcinomas. Expression of IL‐10 protein was higher in prostatic carcinoma and PIN compared to normal prostatic tissue adjacent to the tumor [40]. Another study showed IL‐10 upregulation in prostate cancer compared to benign prostatic hyperplasia [41]. IL‐10 expression level increased with prostate cancer stage and metastatic status [41]. On the other hand, circulating level of IL‐10 was associated with reduced risk of prostate cancer in a cohort of elderly men [42]. Exact contribution of IL‐10 suppression in prostate cancer prevention by SFN remains to be investigated. IL‐12 has antitumor effect in prostate cancer [43]. A single intratumoral injection of a recombinant adenovirus expressing murine IL‐12 resulted in suppression of prostate tumor growth and suppression of lung metastasis due to elevation of natural killer cell‐mediated cytolysis and nitric oxide synthase activity [43]. IL‐12 gene‐modified bone marrow cell therapy inhibited metastatic prostate cancer in mice [44]. It is possible that SFN‐mediated prevention of prostate cancer is increased by IL‐12 intervention. Ultimately, the observed decrease in plasma IL‐10 and IL‐12 in this study likely reflects a reduction in overall tumor burden and chronic inflammation, rather than SFN‐mediated immunosuppression.

We found that Myc overexpression confers partial but significant protection against SFN‐mediated downregulation of ACC1 and FASN, as well as suppression of plasma TFFA level. Prostate tumors show marked overexpression of *Myc* mRNA and protein, which correlates with increased disease severity [45, 46]. Because SFN‐mediated suppression of TFFA was only partially protected by Myc overexpression, additional mechanisms are likely to explain these results. Sterol regulatory element‐binding protein 1 plays an important role in fatty acid synthesis [47]. We have shown previously that SFN treatment decreases protein level of sterol regulatory element‐binding protein 1 in cultured prostate cancer cells [24]. Thus, it is reasonable to conclude that suppression of TFFA level by SFN administration is mediated by downregulation of both Myc and sterol regulatory element‐binding protein 1.

In conclusion, the present study reveals that prostate cancer prevention by oral administration of SFN is associated with suppression of fatty acid synthesis in Hi‐Myc mice. To establish the translational merit of our mouse study, we conducted a randomized and placebo‐controlled clinical trial of SFN‐rich dietary supplement BroccoMax in prostate cancer patients scheduled for prostatectomy [48]. Four‐week treatment with BroccoMax resulted in a significant decrease in prostate tumor expression of ACC1 and FASN when compared to the placebo group [48]. However, BroccoMax intervention did not reduce circulating or prostate tumor levels of TFFA [48]. There could be two reasons why TFFA was not decreased: (a) these patients with advanced prostate cancer (T3 or higher) likely had elevated levels of TFFA; and (b) a treatment duration of > 4 weeks may be required to significantly decrease blood TFFA level. For example, patients with elevated serum cholesterol are advised to get tested 3–6 months after the start of statin therapy. Treatment duration of > 4 weeks was not possible in our clinical study because patients would not agree to delaying surgery for an experimental study.

## Author Contributions

Krishna B. Singh and Eun‐Ryeong Hahm performed experiments, analyzed data, and wrote the first draft of the manuscript. Bruce L. Jacobs and Shivendra V. Singh conceived and/or supervised the study. All authors reviewed and approved the final manuscript.

## Ethics Statement

Use of transgenic FVB‐Tg (ARR2/Pbsn‐MYC) (Hi‐Myc) mice in this study was approved by the Institutional Animal Care and Use Committee of the University of Pittsburgh (protocol #14033214).

## Conflicts of Interest

The authors declare no conflicts of interest.

## Data Availability

Data generated from this study are available from the corresponding author on reasonable request.
